# A Systematic Review of Sensor-Based Methods for Measurement of Eating Behavior

**DOI:** 10.3390/s25102966

**Published:** 2025-05-08

**Authors:** Delwar Hossain, J. Graham Thomas, Megan A. McCrory, Janine Higgins, Edward Sazonov

**Affiliations:** 1 Department of Electrical and Computer Engineering, University of Alabama, Tuscaloosa, AL 35401, USA; dhossain@crimson.ua.edu; 2 Weight Control and Diabetes Research Center, The Miriam Hospital, Providence, RI 02903, USA; john_g_thomas@brown.edu; 3 Department of Psychiatry and Human Behavior, Warren Alpert Medical School of Brown University, Providence, RI 02903, USA; 4 Department of Health Sciences, Boston University, Boston, MA 02215, USA; mamccr@bu.edu; 5 Department of Medicine, University of Colorado Anschutz Medical Campus, Aurora, CO 80045, USA; janine.higgins@childrenscolorado.org

**Keywords:** eating behavior, dietary intake, sensor, technology, wearable sensor, meal microstructure

## Abstract

The dynamic process of eating—including chewing, biting, swallowing, food items, eating time and rate, mass, environment, and other metrics—may characterize behavioral aspects of eating. This article presents a systematic review of the use of sensor technology to measure and monitor eating behavior. The PRISMA 2020 guidelines were followed to review the full texts of 161 scientific manuscripts. The contributions of this review article are twofold: (i) A taxonomy of sensors for quantifying various aspects of eating behavior is established, classifying the types of sensors used (such as acoustic, motion, strain, distance, physiological, cameras, and others). (ii) The accuracy of measurement devices and methods is assessed. The review highlights the advantages and limitations of methods that measure and monitor different eating metrics using a combination of sensor modalities and machine learning algorithms. Furthermore, it emphasizes the importance of testing these methods outside of restricted laboratory conditions, and it highlights the necessity of further research to develop privacy-preserving approaches, such as filtering out non-food-related sounds or images, to ensure user confidentiality and comfort. The review concludes with a discussion of challenges and future trends in the use of sensors for monitoring eating behavior.

## 1. Introduction

Eating is a complex interaction of various physiological, emotional, social, cultural, environmental, and economic factors that influence the timing of an eating episode, the amount of food intake, food choices, and when, where, and how food is consumed.

Dietary intake refers to the types and amounts of food and drinks a person consumes over a given period, such as a day or a week. It can be measured in terms of the energy content of food, macronutrients (such as carbohydrates, protein, and fat), micronutrients (such as vitamins and minerals), and other nutritional components. Eating behavior, on the other hand, refers to the way a person eats, including their habits, patterns, and attitudes toward food. Eating behavior encompasses factors such as meal timing, portion sizes, food selection, eating speed, social context, and emotional influences on eating. While dietary intake and eating behavior are related, they remain distinct concepts. A person’s dietary intake is influenced by their eating behavior, but eating behavior is shaped by various factors beyond just the types and amounts of food consumed. Understanding both dietary intake and eating behavior is crucial for developing effective strategies to promote healthy eating habits and overall well-being. This review focuses on eating behavior.

Eating behaviors (i.e., food choices, motives, and feeding practices) play a crucial role in the development of chronic diseases, including type 2 diabetes, heart disease, and obesity [1,2,3]. As such, ongoing research focuses on the automatic detection of eating episodes, the recognition of consumed foods, and the measurement of food quantity and manner of consumption. One approach to understanding the complexity of eating behavior is to track micro-level temporal patterns within an eating episode, such as biting, chewing, swallowing, food items, eating duration, eating speed, consumed mass, and eating environment. This review explores the literature on sensor modalities and other technologies used to identify and monitor these eating behavior measures.

Historically, dietary intake assessments in adult populations have relied on self-report methods [4,5], such as 24-h recalls, food records (food diaries), and food frequency questionnaires (FFQs) [6,7]. While these methods help establish relationships between eating behavior and dietary intake, they fail to capture important eating behavior measures and processes. Many aspects of eating behavior cannot be accurately assessed through self-reporting due to the lack of granularity in measuring food consumption and the subconscious nature of repetitive eating actions [8].

Rapid advancements in technology have enabled the development of accurate and objective systems for measuring eating behavior with fine granularity. Most technology-based eating behavior monitoring systems rely on sensors, including acoustic, motion, distance, strain, physiological, and image (camera) sensors. Wearable sensors include devices placed on the head or neck to detect chewing or swallowing [9,10,11,12,13,14] and wrist-based inertial sensors to track hand-to-mouth gestures as a proxy for bites [15,16]. Camera-based methods [17,18,19,20,21,22,23] use food images, typically captured before and after an eating episode, to recognize consumed food items and estimate energy intake (EI). The availability of image-capturing devices has allowed researchers to analyze what people eat during an eating episode. Image capture can be categorized into two methods: active (manual capture, typically before and after the eating episode) and passive (wearable cameras that continuously capture images or do so at pre-determined intervals). The captured images are then analyzed manually by nutritionists [20,21] or through computer vision algorithms [22,23].

The primary objective of this review is to systematically evaluate sensor-based methods for measuring various aspects of eating behavior, including biting, chewing, swallowing, food selection, consumed mass, energy intake, eating speed, and eating environment. Each eating behavior measure (e.g., chewing) can be quantified using different metrics (e.g., number of chews, chewing rate/frequency). Most existing review papers, such as [24], primarily focus on detecting eating events and dietary intake rather than the broader domain of eating behavior. For instance, computer vision and wearable sensor-based approaches for eating detection have been explored in reviews [24,25,26,27]. A few reviews have focused on specific eating behavior metrics, such as chewing and swallowing [28], energy intake [29,30], and food portion size [31]. Currently available methods to automatically detect eating behavior events, such as bites, chews, and swallows, from only video recordings using cameras were reviewed in [32]. Another literature review [33] focused on both eating/drinking detection and the detection of eating behavior metrics following the identification of eating or drinking events, including metrics like bites, chewing, swallowing, and portion size in real-life settings only. However, to develop a comprehensive taxonomy of technologies and the range of eating behavior metrics, it is important to include other studies relevant to aspects of eating behavior, such as eating rate and eating environment, in both laboratory and real-life settings. In this review, we aim to fill that gap by systematically reviewing the sensors and methods used for monitoring eating behavior in both laboratory and free-living settings by providing a comprehensive taxonomy of measured eating behavior metrics and technology used. Our search was conducted across five major databases and followed the Preferred Reporting Items for Systematic Reviews and Meta-Analyses (PRISMA) guidelines [34]. The contributions of this review are twofold: (i) We propose a taxonomy of quantifiable eating behavior metrics and corresponding sensors and measurement devices. (ii) We evaluate the accuracy of these measurement devices and their applicability in free-living conditions.

The paper is structured as follows: Section 2 presents the methodology of the systematic review, including research questions (RQs), the search strategy, and database search results. Section 3 discusses the review findings and taxonomy, with a detailed description of selected articles. Section 4, Section 5 and Section 6 cover discussions, challenges, future directions, and the conclusion, respectively.

## 2. Review Methodology

The systematic review protocol was developed following the Preferred Reporting Items for Systematic Review and Meta-Analysis (PRISMA) 2020 guidelines. The titles and abstracts of the publications retrieved through the database search were independently screened by two of the authors, and then the full-text review of all relevant studies was carried out. The review was not registered, and a protocol was not prepared. The following processes were used in this methodology:

### 2.1. Research Questions Identification

Two research questions were identified to guide this systematic review.

RQ1: What quantifiable metrics of eating have been measured by sensor-based devices to describe eating behavior?

RQ2: What state-of-the-art sensors and other technologies have been employed to measure these quantifiable eating behavior metrics?

### 2.2. Databases

Exhaustive electronic searches for relevant literature were performed across five databases: ACM digital library, IEEE Explore, SCOPUS, PUBMED, and Science Direct from inception through 30 January 2025.

### 2.3. Search Strategy

To construct a comprehensive and systematic search strategy, we began with an initial exploration of the literature using broad terms related to ‘eating behavior’, ‘dietary habits’, and ‘food intake’. This helped us gain an overview of the commonly used language in the field. We then extracted frequently occurring keywords from the titles, abstracts, and keyword sections of relevant articles. This process was supported by tools such as PubMed’s MeSH terms. The resulting preliminary list of keywords was reviewed and refined in consultation with domain experts to ensure that the selected terms accurately reflected key concepts in the study of eating behavior. The following keywords were considered: ‘chewing’, ‘chewing rate’, ‘chewing frequency’, ‘biting’, ‘bite rate’, ‘bite frequency’, ‘swallowing’, ‘swallowing rate’, ‘swallowing frequency’, ‘eating rate’, ‘eating speed’, ‘eating duration’, ‘mealtime’, ‘food items’, ‘portion size’, ‘eating amount’, ‘eating environment’, ‘sensor’, ‘device’, ‘technology’.

To ensure comprehensive coverage of the literature related to sensor-based monitoring of eating behavior, we developed three search queries that shared a common sensor-related component—(sensor OR device OR technology)—and combined them with different categories of eating behavior metrics. These categories included general behaviors (e.g., chewing, biting, swallowing, food item, eating environment), frequency/rate-based measures (e.g., chewing rate, chewing frequency), time and speed of eating (e.g., mealtime, eating speed). Although these queries share a common structure and could be logically merged, we presented them separately to demonstrate our structured approach in covering diverse aspects of eating behavior.

Three query strings were formed to use for the search. The search query strings are as follows:

1. (chewing OR biting OR swallowing OR food items OR eating environment OR portion size) AND (sensor OR device OR technology)
2. (chewing rate OR chewing frequency OR bite rate OR bite frequency OR swallowing rate OR swallowing frequency) AND (sensor OR device OR technology)
3. (mealtime OR meal duration OR eating duration OR eating rate OR eating speed) AND (sensor OR device OR technology)

These queries could alternatively be expressed as a single combined string for implementation efficiency in databases that support complex Boolean logic (where* represents a wildcard)

(chew* OR (chew* AND (rate OR frequency)) OR bite* OR (bite* AND (rate OR frequency)) OR swallow* OR (swallow* AND (rate OR frequency)) OR (eating* AND (amount OR speed OR rate OR duration OR environment)) OR portion size OR food item OR mealtime OR meal duration) AND (sensor OR device OR technology). This alternative format was validated to yield equivalent results to the segmented queries when tested in other databases [e.g., PubMed, ACM], ensuring consistency and reproducibility of the search process.

### 2.4. Inclusion and Exclusion Criteria

The search results were restricted to English-language publications. References from the selected primary full-text articles were further analyzed to identify additional relevant studies. The selection was then refined by applying the eligibility criteria outlined in Table 1 to exclude irrelevant articles. These criteria were established to ensure the inclusion of high-quality, peer-reviewed studies that provide quantifiable metrics on eating behavior while excluding non-relevant, non-English, and non-peer-reviewed sources. A manual bibliographic search was also conducted to identify articles from sources outside the mentioned databases, such as reference lists of the selected articles. Articles that met the inclusion criteria were considered for this review, while those that met the exclusion criteria were filtered out.

### 2.5. Results

**Figure 1:**
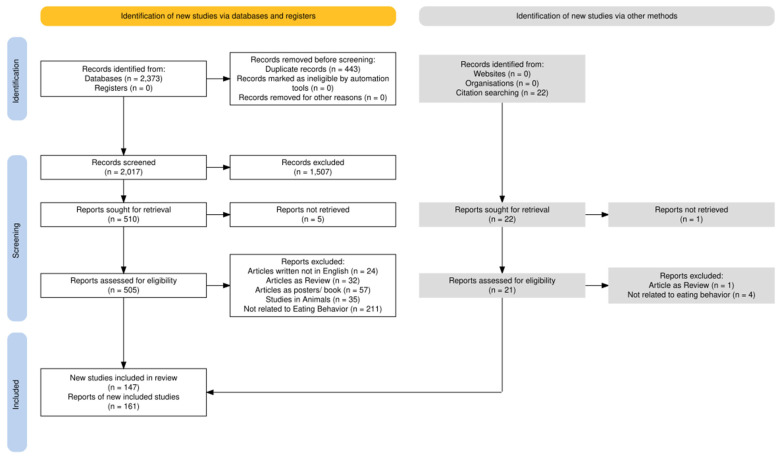
Flow chart outlining the study selection process following the PRISMA 2020 guidelines, made using the R ShinyApp [35].

The initial electronic database searches resulted in a total of 2373 publications. Table 2 shows the number of identified publications from each database for each search string. A manual bibliographic search also identified 22 additional publications that qualified for inclusion. After removing the duplicate articles, a total of 2017 articles were set aside for screening based on abstracts and titles; of these, 505 articles were selected for full-text review. Following the exclusion criteria, 161 articles were included in this systematic review based on the PRISMA guideline process. The methodology and results of the review process are illustrated in Figure 1.

## 3. Review Findings

### 3.1. Taxonomy

**Figure 2:**
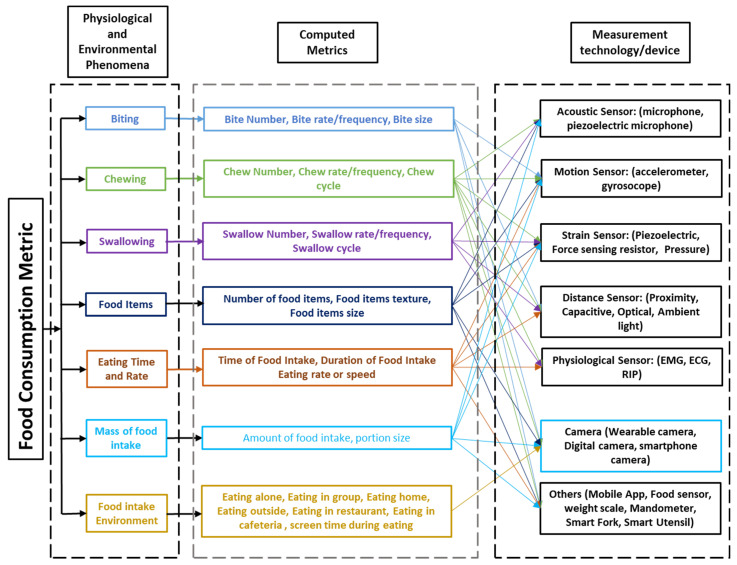
Taxonomy of quantifiable eating behavior metrics and corresponding sensors and measurement devices.

First, the taxonomy of quantifiable metrics that are being measured by sensors to describe the physiological and environmental phenomena of eating behavior was identified to address RQ1. Then, the specific sensor modalities and other technologies that are being used to measure the eating behavior metrics were identified to address RQ2. A classification scheme based on quantifiable metrics to describe different measures of eating behavior using technology is proposed. Each eating behavior measure can be represented using different metrics that can be measured utilizing different sensor modalities and other technologies. A taxonomy that was established from the findings of this review is presented in Figure 2.

### 3.2. Physiological/Environmental Phenomena and Computed Metrics

We identified the physiological and environmental phenomena of eating behavior and grouped them into seven major classes related to biting, chewing, swallowing, food items, eating time and rate, the mass of food intake, and the food intake environment. Each eating behavior measure is described in detail below.

#### 3.2.1. Metrics Related to Biting

Biting is recognized as the initial stage of food ingestion [36] and involves two main actions: the movement of the wrist and hand to transport food to the mouth, and the activation of the jaw muscles to take a bite. Researchers have investigated several metrics of biting behavior during eating events, such as the number of bites [37,38,39,40], bite rate or frequency [41], and bite size [42], to measure the eating behavior. The number of bites refers to the count of individual instances of taking the food by the person using a straw, glass, bottle, spoon, fork, or hand [40]. This metric is often studied in the context of eating behavior and portion control, providing insights into consumption patterns and their potential impact on overall food intake [37,43,44]. Bite rate or frequency reflects the pace and rhythm of eating, which can influence energy intake; for example, a slower bite rate is often associated with reduced energy intake [41]. Bite size refers to the amount of food taken in a single bite, typically measured in grams [43]. Studies have shown that larger bite sizes can lead to increased food consumption, contributing to overeating and potential weight gain [42]. Feedback from these biting metrics—such as bite count, rate, and size—can significantly affect the total amount of food and energy consumed [37,41,42,43,44].

#### 3.2.2. Metrics Related to Chewing

Chewing plays a crucial role in the physical breakdown of food. During chewing, the process forms a bolus, which is later swallowed and moved down the esophagus to the stomach. Researchers have studied various aspects of chewing behavior, such as chewing cycles or bouts [45,46,47], the number of chews [40,48], and chewing rate or frequency [49,50] to better understand eating behavior. A chewing cycle or bout refers to a repetitive sequence of jaw movements and muscle contractions involved in breaking down food, and it influences eating behavior by affecting food processing efficiency, eating pace, and overall satisfaction [45]. The number of chews refers to the count of masticatory movements taken while consuming food [40]. Chewing rate or frequency measures the speed of these movements, typically expressed in chews per minute [49]. These parameters, including the number of chews, chewing cycles or bouts, and chewing rate or frequency are closely related to eating behavior. These parameters—chewing cycles, the number of chews, and chewing rate—are all closely linked to eating behavior. Variations in these factors can impact satiety, portion control, nutrient absorption, and the overall enjoyment of eating, which in turn can affect dietary habits, digestion, and health outcomes [51,52].

#### 3.2.3. Metrics Related to Swallowing

Swallowing is a vital component of eating behavior as it facilitates the movement of food from the mouth to the stomach, enabling the body to digest and absorb essential nutrients. The speed and efficiency of swallowing can significantly influence the overall digestive process and the body’s ability to absorb nutrients effectively [53]. Therefore, understanding the mechanics of swallowing and its role in digestion is a key aspect of studying eating behavior. Researchers have examined various characteristics of swallowing during eating events, such as the swallowing cycle, the number of swallows, and the swallowing rate or frequency, to better describe this dynamic process [12,53,54,55,56]. The swallowing cycle refers to the coordinated sequence of muscle movements and reflexes involved in transferring food or liquid from the mouth to the stomach [53]. The number of swallows denotes how many times a person swallows while consuming food or beverages [12]. Swallowing rate or frequency measures the speed of these actions, typically expressed in swallows per minute [56]. These metrics offer valuable insights into eating pace, potential risks of overeating, and the overall efficiency of the swallowing process. Such information can be particularly useful for individuals aiming to manage their dietary habits, whether by controlling portion sizes or addressing specific swallowing difficulties.

#### 3.2.4. Metrics Related to Food Items

The type and variety of food items can serve as important indicators of eating behavior, offering valuable insights into an individual’s dietary habits and patterns. Researchers have examined various metrics related to consumed food items, including the number of food items, food item types, and textures to better understand the dynamic process of eating [57,58,59,60,61]. The foods an individual chooses to consume can reflect their eating habits, personal preferences, cultural influences, and attitudes toward food.

#### 3.2.5. Metrics Related to Eating Time and Rate

Eating time and rate are key aspects of eating behavior that help characterize the dynamic process of eating [62,63,64,65,66]. Eating duration refers to the length of a meal or snack, measured from the first bite to the last [67]. A prolonged eating duration may be linked to greater meal enjoyment but can also contribute to overeating and poor dietary habits. In contrast, shorter eating durations are often associated with increased feelings of fullness and improved portion control [68]. Eating rate or speed refers to how quickly an individual consumes a meal or snack, typically measured in biting, chewing, and swallowing rate or frequency [69]. Eating too quickly can lead to overeating, as the brain may not have enough time to register feelings of fullness before excess food is consumed. Conversely, eating too slowly has been linked to disordered eating behaviors, such as anorexia nervosa [70].

#### 3.2.6. Metrics Related to the Mass of Food Intake

The mass of consumed food is a significant variable that researchers have explored to better understand the dynamic process of eating. Metrics related to food mass, including the amount and portion size of consumed items, have been used to track eating behavior [61,71,72,73,74]. The amount of consumed food refers to the total weight of food and beverages ingested during a meal or eating episode, typically measured in grams. Food mass can be measured through continuous monitoring of the weight or quantity of food items [75], by using a body-worn sensor [71], or from images using computer vision or a deep learning algorithm [61]. Monitoring these measures provides valuable insights into portion control, nutrient intake, and the balance between caloric and non-caloric components of a diet, which is essential for understanding and managing eating behaviors [76]. Portion sizes can be expressed as mass (g), volume (ml), household measures (e.g., tablespoons), hand measures (e.g., a fist), or as measures relative to the size of a reference object (e.g., “tennis ball”) [77]. Consuming large food portions has been linked to increased energy intake (EI) and a higher risk of adiposity [78].

#### 3.2.7. Metrics Related to the Environment of Food Intake

The environment in which an individual consumes food is a significant factor influencing eating behavior. This environment encompasses various elements, including physical surroundings, social settings, and cultural context [79]. The physical environment refers to factors such as lighting, temperature, noise levels in the dining area, and the type and quantity of food available [80]. The social setting also plays a crucial role, as eating in the presence of others can affect food choices, portion sizes, and eating speed [81]. Overall, the eating environment significantly impacts an individual’s dietary decisions, portion control, and eating pace. In this review, we identified environmental metrics, such as whether an individual eats alone or in a group, at home or in a restaurant, and the amount of screen time during meals [81,82,83].

### 3.3. Measurement Devices/Sensors

Rapid advancements in technology have provided the tools to develop accurate and objective systems for measuring physiological and environmental phenomena related to eating behavior. In this review, measurement devices are grouped into seven major categories based on the integrated sensor system. The seven major groups are (i) Acoustic sensors (microphones); (ii) Motion sensors (e.g., accelerometers); (iii) Strain sensors (e.g., piezoelectric, force sensing resistor, pressure); (iv) Distance sensors (proximity, capacitive, optical); (v) Physiological sensors (EMG—Electromyogram, ECG—Electrocardiogram, RIP—Respiratory Inductance Plethysmography); (vi) Cameras; and (vii) Others (mobile application, weight scale, smart fork, smart utensil). Measurement devices for each quantifiable eating behavior metric found in this review are described below. Most of these measurement devices can be used for assessing both dietary intake and eating behavior. For dietary intake monitoring, these devices are typically designed to measure the nutritional content of the food, such as energy, macronutrients (carbohydrates, fats, and proteins), and micronutrients (such as vitamins and minerals). In this review, we focused on the use of measurement devices for eating behavior.

#### 3.3.1. Biting

##### Motion Sensors

Motion sensors can effectively monitor eating behavior by tracking movements of the head, mouth, jaw, and wrist. Researchers have explored the automatic detection of eating behavior by identifying an upward, arcing motion from the table to the mouth, known as a wrist roll. In one study, an orientation sensor (InertiaCube3) was placed on participants’ wrists to analyze wrist-rolling motions related to biting behavior [38]. Validation was conducted with ten participants who consumed a meal of their choice using utensils or fingers in a controlled laboratory setting. The device demonstrated a 91% sensitivity in detecting bites compared to manual video annotations. Using the acceleration data from a sensor placed on the wrist of the user during a meal, a real-time food intake monitoring system for mobile devices was introduced [84]. This system provided real-time feedback on eating trends, including the total number of bites and bite rate per minute. A tri-axial accelerometer was used to detect wrist rolls, achieving an 81.2% accuracy in bite detection during a controlled experiment involving 15 adults, with observations recorded through a one-way mirror [39]. The Bite Counter seemed challenging to use while eating naturally as an individual had to use similar wrist movements each time he or she took a bite. A wrist-worn bite counter using a gyroscope was described in [44], detecting distinct hand-to-mouth motion patterns typical of food and beverage intake. Real-time feedback on bite count and rate was provided during food consumption. Another wrist-mounted device featuring accelerometer and gyroscopic sensors was introduced in [85] as a bite counter device. In a study involving three participants who wore the device for a full day in free-living conditions, the system achieved a 91.8% accuracy in counting bites, validated against manual video annotations from a GoPro camera positioned on the chest. A food journaling system named Annapurna, ref. [86], runs on a smartwatch and utilizes accelerometer and gyroscope data to identify eating gestures and capture food images. A laboratory study involving 21 participants and 131 eating episodes demonstrated a 6.5% false positive rate and a 3.3% false negative rate in bite detection. Another study [87] proposed using an accelerometer and gyroscope to track wrist motion during meals, involving 271 participants consuming 374 food and beverage items. The system achieved a sensitivity of 75% and a positive predictive value of 89% compared to manual video annotation. The same device was used to develop a deep-learning neural network [88] for segmenting and classifying eating gestures based on wrist motion, achieving an average recognition accuracy of 79.7% per meal. In [89], inertial signals from a smartwatch were used to detect hand-to-mouth gestures and identify food intake events, achieving an F1 detection score of 0.91 in a study involving 21 meals from 12 participants. From in-the-wild collected inertial data (acceleration and orientation velocity) using a smartwatch [90], a complete framework for automatically measuring eating behavior by wrist roll was proposed. The proposed algorithm achieved a 0.92 F1 score in the detection of the bites in free-living conditions against manual annotation from video in leave-one-subject-out (LOSO) validation from 12 subjects. An approach was introduced in [91] to use commercial smartwatch inertial data to estimate the bite size as well as the weight of a bite. The proposed approach was tested against the publicly available dataset which contains smartwatch inertial data from 10 participants, with manually annotated start and end times of each bite along with their corresponding weights from a smart scale, under semi-controlled conditions. Under a leave-one-subject-out cross-validation scheme, the proposed approach achieves a mean absolute error (MAE) of 3.99 g per bite.

##### Distance Sensors

Distance sensors can be used to detect biting behavior by measuring the distance between the user’s hand and mouth or between a necklace and jaw. Various types of distance sensors, such as proximity sensors, infrared sensors, and laser distance sensors, can be employed for this purpose. While each sensor type uses different technology, they all operate on the same principle: the sensor emits a signal that reflects off an object (e.g., the mouth, jaw, or temporalis muscle), and the time it takes for the signal to return is measured. A magnetic-field-based approach for detecting hand movements during biting, using a wearable device called eButton, was presented in [92]. In this method, a miniature magnetic marker is worn on a finger, while a magnetometer embedded in the eButton—worn on the chest detects signals generated by eating-related hand movements. Analyzing these signal patterns helps differentiate eating from other daily activities, reducing false detection rates. Additionally, an ambient light sensor integrated into a multi-sensor necklace prototype for eating detection was introduced in [93]. This sensor detects a drop in light levels when the individual’s hand moves toward the mouth during a feeding gesture, allowing for accurate detection of biting instances. Data collected from 10 participants (five obese and five with normal weight) over two days in free-living conditions showed that the device achieved an accuracy of 82.2% in counting bites, compared to manual video annotation.

##### Cameras

Eating videos recorded with a digital camera in a profile view were used to automatically count the number of bites using a combination of object detection, image classification, and the affine optical flow algorithm [40]. This algorithm achieved an accuracy of 85.4% ± 6.2% when tested on 84 meal videos from 28 participants in a laboratory setting. A method utilizing skeletal features from videos to automatically measure meal duration, bite rate (or frequency per minute), and the number of bites was presented in [94]. The proposed Rapid Automatic Bite Detection (RABiD) algorithm, which extracts and processes skeletal features, was trained on a dataset comprising 85 meals from 59 individuals across three different foods. When tested on an independent eating behavior experiment involving 18 female participants and 45 meals, the algorithm achieved an impressive F1 score of 0.948 compared to manual annotation. In [95], a depth camera from a Kinect Xbox One tracked the motion of a person’s gesture and posture during food intake to detect and report the biting instances. The proposed system achieved an accuracy of 96.2% for counting the number of bites against manual annotation in the laboratory setting for 10 meals from 10 volunteers. In [96], authors proposed a vision-based approach to capture shared food consumption with a 360-degree camera placed in front of the participants, then used a neural network to infer different eating states. The authors reported a bite detection error of 26.2%. A wearable fish eye camera was used in [37] to record 16 participants’ free-living eating behaviors, detect biting events, and report the number of bites and bite rate/frequency per minute as eating behavior. A video dataset collected by recording the meals of 264 participants in a restaurant was described in [97]. Participants consumed 374 different types of intake. The camera was installed on the ceiling of the restaurant to minimize the effect of an experimental observer. Three state-of-the-art models, namely CNN-LSTM, SlowFast, and X3D-L, were used to explore the feasibility of detecting intake gestures using the dataset and establishing baseline performance. Results indicate that the best F1 score was 0.899 in biting gesture detection against manual annotation. To measure eating behavior from videos, a rule-based system to count bites automatically with 468 3D facial key points was proposed in [98]. The proposed approach achieved 79% accuracy in counting the number of bites against manual annotation in 164 videos from 15 participants. A framework allowing a global detector to learn meal-length patterns with manageable computational demands, with a new augmentation technique to generate hundreds of meal-length feature samples per video, facilitating effective training of a global detector with limited video availability was introduced in [99]. On the Clemson Cafeteria dataset of 486 meal videos, the proposed method achieves F1 score of 0.93 for bite gestures against manual annotation.

##### Others

An augmented fork was proposed In [100], a 15-week study including 141 obese participants proposed using an augmented fork to deliver real-time feedback based on the participants’ average bite rate per minute. The findings of this study indicated that an augmented fork with vibrotactile feedback was a viable tool to reduce bite rate in naturalistic settings. However, the study excluded daily dietary and nutritional intake measures to avoid overburdening participants, as repeated food reporting could interfere with natural behavior and compromise the validity of the intervention. Because of this, the study offered limited insights into food intake and satiety levels. A tool in the form of a plate system that automatically measured the amount per bite in grams during eating was proposed in [101]. Weight sensors were integrated into the base, allowing the plate to be easily removed and cleaned. Data from 24 adults (ages 52–95) eating a single meal with chopsticks were used to train and evaluate the model. Out of a total of 836 true manually annotated bites, the algorithm detected 602 bites with a precision and recall of 0.78 and 0.76, respectively.

A summary of the measurement devices found in this review for metrics related to biting is provided in Table A1.

#### 3.3.2. Chewing

##### Acoustic Sensors

A wireless in-ear microphone prototype using a Bluetooth headset was developed [102] to detect chewing sounds. A two-stage algorithm—chew-like signal detection followed by chewing verification—was implemented to improve accuracy. A wearable sensor consisting of a microphone and a camera placed on an over-the-ear headphone was presented in [103], which autonomously provides detailed information regarding a participant’s dietary habits. When a subject wears the sensor, the camera is directed towards the table to take images of the food container. The microphone is placed just outside the ear canal to measure sound propagation. Sound features are extracted in real-time, and if a chewing activity is classified, the camera captures a video sequence for further analysis. The proposed system reported 80% accuracy against manual annotation in counting the number of chews using data from six volunteers consuming lunch in a university restaurant. The applicability of the sensor in real-world scenarios was further examined through a user feedback survey. The results indicated that, while the comfort level received only average ratings, the device used was a modified off-the-shelf product that was not originally designed with user comfort in mind. Microphones placed in the outer ear canal were used in [10] to detect chewing sounds during eating. Eight algorithms were evaluated on two datasets containing 68,094 chews across approximately 18 h of recordings, achieving over 80% precision and recall against manual annotations. However, the sensor system could be used to analyze the chewing frequency of people only in laboratory settings. An embedded hardware prototype that precisely records acoustic signals during eating in a noninvasive manner using a high-fidelity throat microphone worn on the neck was presented in [104]. Using Hidden Markov Models for detection, the system reached an accuracy of 86.6% in counting chews across 12 volunteers in a laboratory setting. A survey regarding wear comfort and functionalities of the sensor system was conducted. The results showed that the current design of the sensor system was acceptable to most users for daily use. A throat microphone-based system, Audacity, was explored in [105] to detect chewing cycles in noisy restaurant environments. Combining clean chewing recordings with background noise at a −10 dB signal-to-noise ratio, the system achieved an F1 score of 71.4% across 12 participants, with chew events manually annotated through visual and audio inspection using Audacity. Off-the-shelf Bluetooth headsets were used [106] to monitor chewing episodes through sound analysis unobtrusively. A Support Vector Machine (SVM) model achieved 94–95% accuracy in laboratory settings but dropped to 65–76% in real-world scenarios. Earbud microphones paired with audio sensors were used in [107] to recognize eating gestures in real-time across 12 participants. A laryngeal throat microphone was used [108] to develop a chewing detection method based on recorded food intake sounds during meals. The system achieved accuracies of 0.712 and 0.783 for participant-independent and subject-dependent settings, respectively, among eight participants in laboratory settings. A deep-learning-based classification method improved accuracy to 77–94% despite ambient noise. A bone conduction microphone combined with a smartphone was proposed in [109] to collect intra-body sound signals for feedback on chewing rate and frequency. Using a medium Gaussian SVM (RBF kernel), the system achieved 97.6% accuracy in counting chews. Bone conduction microphones capturing chewing sounds combined with additional background noises were explored in [110], achieving 97.1% accuracy using a medium Gaussian SVM in controlled settings. An ear-worn device, eSense was investigated in [111] for detecting chewing activities based on audio and inertial sensor data. The results of this study, which included five volunteers, indicated that fusing audio and inertial sensor modalities improved the accuracy to 97% (compared to 95% using inertial data alone) in counting the number of chews against manual annotation from video in laboratory settings. A skin-contact microphone, placed behind the ear to capture chewing sounds, was tested [112] and achieved an F1 score of 84.5% in data collected from 10 participants in laboratory settings. A wearable device equipped with a two-channel condenser microphone was proposed in [113] for chewing detection. Tested on 18 participants consuming gum, crackers (Ritz), and shredded cabbage, the system achieved an F-score of 0.80 against manual annotations in a controlled setting. A custom earbud with two microphones was combined with an Android smartwatch utilizing 9-axis IMU data on each wrist, then used in [114] to introduce Stochastic Variational Deep Kernel Learning (SVDKL). This method classified micro-events (chews) within macro-events (meals), achieving 84% recall and 67% precision across 30 meals from six volunteers in a laboratory setting. The Razer Anzu smart-glass, equipped with stereo speakers and microphones, was explored in [115] for detecting chewing. Using a Support Vector Machine and data from five participants consuming various foods across 12 meals, the system achieved an F-score of 0.96 against manual annotations in laboratory settings.

##### Motion Sensors

An ultra-miniaturized Inertial Measurement Unit (IMU), *WB-3*, was used in [116] for efficient chewing detection through jaw tracking. The WB-3 IMU integrates a 3-axis gyroscope, 3-axis accelerometer, and 3-axis magnetometer, enabling measurement of jaw movement acceleration, angular speed, and mouth opening angle. A 3-axis accelerometer attached to eyeglasses captured food intake data from 10 participants across both laboratory and free-living settings [117]. The proposed approach can provide accuracy comparable to other devices presented in literature without the need to use sensors that require constant contact with the skin. Feature selection with a kNN classifier yielded an average F1-score of 87.9% for 20-s epochs in laboratory and free-living conditions. A single-axis accelerometer attached to the temporalis muscle was used in the CARE system [118] to detect muscle bulges and recognize chewing activity. This system also calculates chewing rate or frequency by identifying periodic acceleration patterns. The system achieved an accuracy of 83.2% compared to manual video annotation in detecting chewing cycles and counting chews among 15 volunteers in laboratory settings. Information provided by the system includes the number of chewing cycles, total chews, and chewing rate or frequency per minute. An accelerometer-based system, IDEA [119] was proposed for accurate eating action identification by detecting chewing. The system achieved an accuracy of 92.1% in counting chews compared to manual video annotation among 36 volunteers in a laboratory setting. Feedback is provided to users three minutes after the start of an eating episode and every two minutes thereafter, based on the computed chewing rate or frequency as an indicator of eating speed. Earphones embedded with Inertial Measurement Units (IMUs) were explored in [120] for unobtrusive chewing detection and counting. The system, named IMChew, uses time and frequency domain features combined with machine learning classifiers to detect chewing and estimate frequency. Data collected from eight participants demonstrated an F1-score of 0.91 for chewing detection and a Mean Absolute Percentage Error (MAPE) of 9.51% for chewing count, indicating strong potential for automated dietary monitoring using IMU-equipped earphones. The ground truth collection method requires participants to press a laptop’s spacebar with each chew, which may introduce bias by causing lower chewing rates.

##### Strain Sensors

A piezoelectric strain gauge sensor [121] was used to capture lower jaw movement for detecting food intake periods through non-invasive chewing monitoring. Data from 20 volunteers during quiet sitting, talking, and food consumption demonstrated a per-epoch classification accuracy of 80.98% using twenty-fold cross-validation. A piezoelectric strain sensor was also utilized in [122] to compare the performance of Support Vector Machine (SVM) and Artificial Neural Network (ANN) classifiers for chewing cycle detection. Time domain (TD) and frequency domain (FD) features were extracted from signals collected over 24 h from 12 participants in free-living conditions. ANN achieved an average accuracy of 86.86% ± 6.5%, whereas SVM (with a linear kernel) achieved 81.93% ± 9.22%. The same piezoelectric strain sensor was used in [48] to record jaw movement during chewing and estimate the number of chews using linear regression. The regression model achieved a mean absolute error of 9.66% across 30 volunteers. A comparison between an off-the-shelf piezoelectric strain sensor and a plotter-drawn strain sensor for quantifying chews across various food items was described in [123]. Data collected from five participants showed absolute mean error rates of 8.09% ± 7.16% and 8.26% ± 7.51% for the piezoelectric and plotter-drawn sensors, respectively. A fully automatic chewing detection system using a piezoelectric strain sensor was presented in [49]. The signal was segmented into 5-s non-overlapping epochs, and an artificial neural network (ANN) was trained using leave-one-out cross-validation. This approach achieved an average F1 score of 91.09% for classifying food intake versus non-intake, with a mean absolute error of 15.01% ± 11.06% for chew counting. A wearable device combining a piezoelectric strain sensor on the temporalis muscle, an accelerometer, and a data acquisition module attached to eyeglasses was developed [124] to detect chewing cycles even during physical activity or talking. Data collected from 10 participants performing activities such as sitting, walking, eating while sitting, and eating while walking resulted in an F-score of 99.85%. In the proposed device, the data collection module was connected to the temple of eyeglasses, which reduced the number of sensors a participant had to wear compared to a multi-sensor system. This could help reduce user burden and increasing user compliance. An Automatic Ingestion Monitor (AIM) sensor system was developed for objective monitoring of ingestive behavior in free-living conditions. Data collected from 12 participants wearing AIM for 24 h demonstrated an average accuracy of 92.1% in counting chews during meals. In [125], AIM was used to test three ensemble classifiers for food intake detection based on chewing, with the Linear Discriminant Analysis (LDA) ensemble using the Bagging technique achieving an average accuracy of 93% compared to manual annotation. A piezoelectric strain sensor placed on the temporalis muscle, integrated into the temple of eyeglasses, was used in [47] to detect and characterize chewing cycles. A multivariate regression model estimated chew counts from classified segments, achieving a mean absolute error of 3.83% on the participant level across data from 10 individuals. A necklace-shaped piezo sensor named Slowee [126] was introduced to detect chewing when worn below the neck. Data from 10 participants in laboratory settings showed an average accuracy of 0.938 compared to manual annotation.

##### Distance Sensors

A wearable computing approach using proximity sensing [127] was introduced to detect chewing and report chewing rate. This system uses a discreet, lightweight instrumented necklace designed to capture head and jawbone movements without direct skin contact. An evaluation conducted with 32 participants across three phases achieved 95.2% precision and 81.9% recall in controlled conditions, and 78.2% precision with 72.5% recall in free-living settings compared to manual annotation by researchers. After the experiment, participants were asked to rate their experience with the device, specifically its comfort level, on a scale from 1 to 5, with 5 indicating the highest level of comfort. The average comfort score reported by the 15 participants was 3.6, with a standard deviation of 0.91. A proximity sensor directed toward the chin [93] was employed on the necklace to monitor signal variations during chewing. Data collected from 10 volunteers in free-living conditions resulted in an F1 score of 77.1% for chewing sequence detection compared to ground truth from video annotations. A small ear-hung wearable device [50] was created to monitor chewing rate using an infrared distance sensor and accelerometer. Worn on the ear pinna, the device recorded chewing cycles, with data transmitted via Bluetooth to a smartphone. Testing on 22 participants in laboratory conditions achieved an accuracy of 88% in counting chews. A proximity sensor-based approach capturing temporalis muscle movement [128] was tested on data from six participants. This system reached a classification accuracy of 97.6% and a chew count error rate of 2.69% compared to manual annotations in laboratory settings. A proximity sensor housed in eyeglasses [129] was utilized to detect chewing by capturing temporalis muscle movements during food intake. Chewing detection was classified using a medium Gaussian Support Vector Machine (SVM), and chew count estimation was optimized using particle swarm optimization (PSO). The validation dataset included 20 participants across eight food types, achieving a detection accuracy of 96.4% and a mean absolute error of 4.26% for chew count estimation against manual annotation.

##### Physiological Sensors

Electromyography (EMG) sensor positioned on the right and left masseter muscles recorded EMG signals during teeth clenching to evaluate chewing behavior [130]. This method achieved an accuracy of 86.4% in counting chews among 56 individuals. Analysis confirmed the role of personality traits on the chewing behavior of the subjects. An EMG sensor used in [131] compared chewing rate or frequency between normal BMI individuals and overweight or obese young adults, and found no significant differences in chewing rate or frequency between 14 high BMI and 14 normal BMI individuals. EMG sensor system described in [126], designed as a headphone-shaped device attached to both sides of the chin, detected chewing with an average accuracy of 0.9 in counting chewing cycles. EMG sensor-equipped smart eyeglasses for dietary monitoring were used in [132], to sense food chewing and report chewing rate or frequency during a study with eight participants eating different foods. EMG sensor testing in a free-living study [133] with the same eyeglasses achieved over 77% precision and recall for chewing detection among 10 volunteers. In-ear microphone combined with a photoplethysmography (PPG) sensor placed in the ear concha was proposed in [134] and achieved up to 0.9 accuracy in counting chews in a laboratory study with 14 volunteers. The EMG sensor placed behind the right ear was tested in [135] to assist self-report methods, demonstrating high sensitivity and specificity (>90%) in detecting chewing in data from 15 volunteers. A Myoware myoelectric sensor was used in [136] to measure the myoelectric potential of the masticatory muscle and develop a system that provided visual and auditory feedback on the number of chews and chewing rate/frequency throughout a meal.

##### Cameras

To achieve a detailed description of chewing patterns throughout a meal, video recordings of the maxillary–mandibular region of women eating from a plate were analyzed using computer vision and deep learning algorithms in [51]. This method was tested on 11 women eating a meal, achieving an accuracy of 73.3% in counting the number of chews against manual annotation in a laboratory setting. Microphone signal collection was used in [103] to detect a chewing sequence, with a camera capturing a video sequence for further analysis. Bite detection from meal videos was explained in [40], which utilized an optical flow algorithm to count chews within detected bites. This chew detection algorithm achieved an accuracy of 88.9% against manual annotation in a laboratory setting. Visual quasi-periodicity in chewing was employed in [137] to detect chewing events and report chewing frequency from videos using support vector machines. The chew counting algorithm achieved 93% accuracy compared to manual annotation from 37 meal videos collected from 37 participants in laboratory settings. A bottleneck of the proposed approach is that it requires a subject-dependent AAM to be trained for each user. In [138], algorithms were designed to determine chew count from video recordings and provide food intake curves, such as variations in chewing frequency and cumulative chew count throughout the meal. An algorithm based on image and signal processing techniques was developed to continuously capture the area of interest from video clips, determine facial landmarks, generate the chewing signal, and process the signal using two methods: low pass filter and discrete wavelet decomposition. Peak detection determined chew count from the output of the processed chewing signal. The system was tested with recordings from 100 participants at three different chewing speeds (slow, normal, and fast), with the low pass filter algorithm achieving the best mean absolute error of 6.48%, 7.76%, and 8.38% for slow, normal, and fast chewing speeds, respectively.

##### Others

To compare chewing strength variation [139], several different sensor modalities, such as pressure sensor, flexible bend sensor, piezoelectric strain sensor, and EMG, were used to measure differences in chewing strength. Results from 15 participants in laboratory setting eating three test foods (carrot, apple, banana) showed that all four of the explored sensor modalities can effectively detect chewing strength variation among test foods. A summary of the measurement devices found in this review for metrics related to chewing is provided in Table A2.

#### 3.3.3. Swallowing

##### Acoustic Sensors

Bone-conduction microphone for non-invasive monitoring of swallowing was presented in [140]. In [12], two methods for acoustical swallowing detection were proposed and compared using sounds collected in [11], which were contaminated by motion artifacts, speech, and external noise. Methods based on the Mel-scale Fourier spectrum, wavelet packets, and support vector machines were studied, considering the effects of epoch size, level of decomposition, and lagging on classification accuracy. The methodology was tested on a large dataset (64.5 h with 9966 swallows) collected from 20 human participants with various degrees of adiposity, achieving an average weighted accuracy of 84.7% in detecting swallowing events compared to manual annotation. Throat and ambient microphones were used in [141] to detect swallowing cycles and report the number of swallows. Data from seven healthy volunteers in laboratory settings achieved an accuracy of 68.2% in counting the number of swallows. The proposed approach detected most swallowing events, but with a high number of false positives caused by chewing and other intrinsic sounds captured by the throat microphones. Precision value of 50% averaged across all subjects may suggest that subsonic frequencies were sensitive to sound artifacts. A throat microphone-based prototype for automated ingestion detection through swallow detection was developed in [142], with data collected from seven volunteers, including different food and drink items. This method achieved 90% classification accuracy in swallow detection against manual annotation in laboratory settings. Swalloscope, a portable wearable system using a neck-worn microphone to detect swallowing in real-time and provide feedback on the number of swallows and swallow rate/frequency, was introduced in [143]. The real-time algorithm for detecting dry and water swallows was based on a template-matching approach. The system achieved an overall accuracy of 79.3% (standard error: 4.2%) in detecting 92 water swallows compared to manual annotation. The device can be used during activities of daily life with minimal intervention, making it potentially more capable of capturing aspirations and risky swallowing patterns through continuous monitoring. In [104], an embedded hardware prototype using a high-fidelity throat microphone worn on the neck collected food intake sensor data to record acoustic signals during eating in a non-invasive manner. Hidden Markov models were used for swallow detection, with data collected from 12 participants consuming seven types of food items. The system achieved 86.6% accuracy in counting the number of swallows. Spectrograms extracted from sound signals recorded by a laryngeal throat microphone were used in [108] with a convolutional neural network (CNN) to detect swallow cycles. The best accuracy results were 0.712 and 0.783 for participants-independent and participants-dependent settings, respectively, among eight participants. In [110], other sounds were added to swallowing sounds recorded by a bone-conduction microphone to create a natural meal environment. Using a medium Gaussian SVM, an accuracy of 97.1% in swallow detection was achieved. In [109], a system composed of a low-cost bone-conduction microphone to collect intra-body sound signals and a smartphone to process them was proposed to support consciousness improvement feedback in real-time and accurate quantified monitoring of swallowing by providing feedback on swallow rate/frequency. Data from six volunteers who wore the device all day achieved an F1 score of 90.2% in swallow detection compared to manual annotation from videos in laboratory conditions.

##### Strain Sensors

A piezoelectric sensor integrated into a wearable necklace was proposed in [144] to detect swallowing by detecting skin motion in the lower trachea during ingestion. An experiment was conducted on 20 subjects eating three types of solid food in laboratory settings, achieving an F score of 86.6% in detecting swallowing events against manual annotation. In [55], integrated wearable necklace comprising two vertically positioned piezoelectric sensors around the neck, an inertial motion unit, and long short-term memory (LSTM) neural networks was presented to detect and count swallows. This system achieved a 3.34 RMSE in swallow count using LSTM and a 76.07% average F-measure of swallow detection compared to manual annotation in a laboratory-controlled study with confounding activities involving 10 participants.

##### Physiological Sensors

A wearable chest belt was used in [145] to detect swallow cycles by differentiating between normal breathing cycles and breathing cycles with swallows. The chest-belt contained a piezo-respiratory belt that converted changes in tension during breathing to a voltage signal. Data were collected from three volunteers who wore the device during one meal, achieving an accuracy of 82.9% in counting the number of swallows after detecting swallow cycles using an SVM classifier. Respiratory Inductance Plethysmography (RIP) belts on the chest and abdomen were used in [146] to detect swallow cycles through breathing signals. Support Vector Machine, estimating the posterior probability, was applied to the extracted features. Experiments on six healthy participants demonstrated that the system achieved a precision of 65.2% and a recall value of 80.3% in detecting swallow cycles against manual annotation. EMG sensors were used in [46] to develop an automated system for detecting swallowing events and providing real-time wristband haptic feedback to facilitate mindful eating. Data were collected from 16 participants eating five different foods in laboratory settings, and 18 features were extracted from the EMG sensor to train a support vector machine classifier. The algorithm achieved an F1 score of 0.87 in swallowing detection in leave-one-subject-out validation against manual annotation.

##### Distance Sensors

Acoustic Doppler Sonar (ADS) was used in [147] to detect swallowing through automatic event recognition via an artificial neural network. A 40 kHz ultrasonic beam was focused on the lower jaw and neck, and the movements of the chin and neck caused Doppler frequency shifts and amplitude envelope modulation of ultrasonic signals. This allowed for the detection of swallow cycles using the Doppler frequency shifts in the received ultrasound signals. Experimental results from ten healthy volunteers in laboratory settings showed that the ADS-based food intake detection method achieved a maximum recognition rate of 78.4% for swallowing detection compared to manual annotation. If food intake is detected solely based on jaw movements, there is a high likelihood that non-eating activities involving jaw motion—such as speaking—will be incorrectly identified as eating events. Therefore, feature variables associated with speech signals were incorporated in this study. A summary of the measurement devices found in this review for metrics related to swallowing is provided in Table A3.

#### 3.3.4. Food Item

##### Acoustic Sensors

A high-fidelity microphone worn on the neck is used in AutoDietary, a wearable system designed to monitor and recognize food intake in daily life [104]. The system includes an embedded hardware prototype that collects sensor data and records acoustic signals during eating in a non-invasive manner. The processed audio data is transmitted via Bluetooth to a smartphone, where a decision-tree-based algorithm identifies food types. In experiments with 12 participants consuming seven different food items—apples, carrots, cookies, potato chips, walnuts, peanuts, and water—AutoDietary achieved an 84.9% accuracy in food-type recognition, and up to 97.6% and 99.7% accuracy in distinguishing liquid and solid foods, respectively, against manual annotation. Another study [148] utilized an audio sensor placed around the neck to classify food types using a random forest classifier. A model with 100 trees was trained on extracted sensor features and evaluated using a Leave One Food Out (LOFO) approach. Testing with 10 participants consuming four food items in a laboratory setting resulted in an F-score of 97.2% for food classification. iHearken, a headphone-like wearable system, used chewing sounds for food type recognition [149]. A bidirectional long short-term memory (BiLSTM) model was applied to classify food types based on the extracted audio signals. Data collected from 16 participants consuming 20 different food items yielded an F-score of 97.4% against manual annotation. The medical significance of this investigation lies in its potential to reliably monitor the clinical development of food intake classification methods through the detection of chewing events in ambulatory (real-world) environments.

##### Motion Sensors

In [71], multi-modal sensing combining in-ear audio and head and wrist motion was used to improve food type classification. Audio features were captured using internal and external microphones in an earbud, while motion data came from 9-axis motion sensors in a smartwatch and Google Glass. A random forest classifier was trained on these features to classify food types. Testing on data from participants consuming 40 different food items showed that the combined sensor approach achieved an accuracy of 82.7%, compared to 67.8% for audio alone and 76.2% for motion data.

##### Strain Sensors

A wearable necklace with an embedded piezoelectric sensor was used to detect skin motion in the lower trachea during ingestion for food type identification [144]. The necklace transmitted data to a smartphone for signal processing and classification. Testing with 20 participants consuming three solid foods (a meat-like veggie patty, mixed nuts, and Snickers bars) and two liquids (room-temperature water and hot tea) showed that the method achieved a precision and recall of 87.0% and 86.3% for liquids and 86.4% and 87.1% for solids, demonstrating effective discrimination between food types. The proposed method achieved an F-measure of 90% in distinguishing between hot and cold drinks and also demonstrated the potential for classifying different solid food types. Expanding the classification to a wider variety of food types and evaluating the system in more naturalistic, free-living environments is the plan of this study.

##### Cameras

In [150], an automatic food image recognition system was developed using the Multiple Kernel Learning (MKL) method to integrate image features such as color, texture, and SIFT. Testing on 50 food categories resulted in a classification rate of 61.34%. A mobile food recognition system [57] performed image recognition directly on a smartphone without requiring server processing, achieving an 81.55% classification rate for the top five category candidates when ground-truth bounding boxes were provided. A food image recognition algorithm [18] was introduced for calorie and nutrition measurement to assist patients and dietitians in managing daily intake, achieving 92.1% accuracy across 15 food items. Graph cut segmentation was used in [151] to enhance food classification accuracy, particularly for mixed foods, improving recognition accuracy by 15% compared to previous work. A real-time eating action recognition system [152] monitored eating behavior using a smartphone, detecting eating moments and classifying food regions near the user’s mouth. The method achieved a 74.8% classification rate among five food items. The MT-Diet demo [153], a fully automated diet assessment system using a smartphone camera, leveraged a database of 244 food items from 80 frozen meals. Food identification using a Support Vector Machine with a Radial Basis Function kernel achieved 88.5% accuracy. MT-Diet was later enhanced [154] by integrating a thermal camera, improving food part isolation accuracy to 97.5% and food type identification to 88.93%. FIT-EVE&ADAM [155] an armband-based diet monitoring system, used electromyogram sensors and color and thermal cameras for food type analysis triggered by a single hand gesture. A Sphere Shaped SVM classifier with the Fuzzy C-Means algorithm [156]. segmented and classified food items, achieving 95% accuracy across 100 food categories. An Android-based food recognition application [157] allowed users to capture food images for classification. A deep learning-based visual food recognition algorithm [158] was tested on two datasets: UEC-100 (100 categories, 8643 images) and UEC-256 (256 categories, 28,375 images), achieving 94.6% and 87% accuracy, respectively. A smartphone-based application [60] recognized 13 types of Thai food using a deep convolutional neural network. Image classification on an Indian food dataset [59] applied transfer learning techniques to 20 food classes, each containing 500 images. Among models tested—InceptionV3, VGG16, VGG19, and ResNet—Google InceptionV3 performed best, with 87.9% accuracy. A deep learning-based food recognition and dietary assessment system [159] analyzed meal images using a three-step algorithm that detected candidate regions and classified objects with a convolutional neural network. Evaluation on UEC-100 and UEC-256 datasets yielded 93.1% accuracy in food recognition.

##### Others

In [160], optical, ion-selective electrical pH, and conductivity sensors were used to classify liquids in a cup. Two experiments demonstrated the feasibility of this approach, achieving up to 79% accuracy across 68 different drinks. Ultrasonic, RGB color, temperature, and accelerometer sensors were integrated into the Liquid Intake Detection System (LIDS) [73] for real-time tracking of fluid intake type and volume. The system included a machine-learning framework for fluid classification, volume estimation, and bottle-state recognition. Extensive experiments collected data across 1200 trials, covering five volume levels, ten fluid types, and three lighting conditions. Results showed fluid type detection accuracy ranging from 74.93% to 94.98% when identifying liquids in unseen bottles. A summary of the measurement devices found in this review for metrics related to food items is provided in Table A4.

#### 3.3.5. Eating Time and Rate

##### Motion Sensors

Using an accelerometer, a real-time food intake monitoring system for mobile devices was developed to provide feedback based on eating speed [84]. Acceleration data from a wrist-worn sensor was transmitted via Bluetooth to a mobile device, where it was analyzed to detect bites and calculate eating rate. A smartwatch app [66] was designed to help users regulate their eating speed. The app measured real-time eating rate and provided feedback to slow down when necessary. Four types of feedback—graphics, text, clock, and vibration—were explored to determine the most suitable option based on the eating environment. IDEA (Instant Detection of Eating Action) [161] was introduced to accurately identify eating actions and provide feedback on eating speed. Using a single wristband with IMU sensors, the system operated without manual intervention. Data collected from 36 participants over meals lasting at least 15 min, with an average of 30 eating actions, showed an eating action identification precision of 0.93. The proposed methodology is plug-n-play and does not need any initialization from the user, hence working in a user-independent manner.

##### Strain Sensors

Strain sensors were used in [48,122,123] to estimate the time resolution required for accurately capturing meal microstructure, including eating rate, meal duration, actual ingestion time, and the number of eating events [65]. Results indicated that sensor-based food intake detection should have a time resolution of ≤5 s for accurate meal microstructure analysis. A necklace-shaped piezoelectric sensor [126] was used to measure meal duration and eating rate based on chewing patterns. A temporal convolutional network combined with a multi-head attention module (TCN-MHA) was developed to detect bites, including eating and drinking gestures, from IMU data. Predicted bite sequences were clustered into eating episodes, and eating speed was calculated by dividing the number of bites by the total eating episode duration. To validate this approach, data from 61 participants, totaling 513 h, were analyzed. Experimental results showed a mean absolute percentage error (MAPE) of 0.110 against manual annotation.

##### Distance Sensors

An earphone-type wearable sensor with a small optical sensor, consisting of a light-emitting diode and a phototransistor, was used to estimate mealtime and eating duration [67]. Inserted into the ear, it measured time variations in received light to determine eating duration. Data were collected from seven participants wearing the device for two hours in free-living conditions. The proposed method accurately detected all mealtimes when compared to self-annotation.

##### Physiological Sensors

A wearable sensor system that monitors breathing signals was used to estimate mealtime and meal duration [162]. Swallowing signatures from breathing signals were combined with hand movement data from an accelerometer to train a hierarchical Support Vector Machine (SVM) classifier and a Hidden Markov Model (HMM). Data collected from 14 participants in laboratory conditions showed that the approach achieved an F-score of over 90%. During data collection, the subjects were asked to press a button for each swallow, which may cause a burden to the user and may alter natural eating behavior. An armband-based diet monitoring system, FIT-EVE&ADAM, used an electromyogram sensor embedded in the armband to track user gestures and estimate eating speed [155].

##### Others

The Universal Eating Monitor (UEM) [64], an apparatus with a concealed electronic balance for continuous weighing of a subject’s plate, was used to explore relationships between self-reported and laboratory-measured eating speed. Coupled with a digital computer, UEM recorded food consumption every three seconds during a single-course meal of a homogeneous food mixture. The effect of eating speed on energy intake in normal-weight and overweight/obese individuals was examined using UEM [63]. The Sussex Ingestion Pattern Monitor was used to study the effect of portion size on bite size, eating speed, deceleration rate, and meal duration [42]. A smart fork designed to provide real-time feedback on eating speed was assessed for acceptability, perceived efficacy, and user experience in a qualitative study [163]. Glucose sensors were used to develop algorithms for eating detection and meal or snack-size estimation [164]. An augmented fork delivering real-time vibrotactile feedback to regulate eating speed was evaluated [165]. A portable sensor sheet with embedded pressure sensors was developed to measure eating pace, time, order, and intake [166]. Designed for home use, it allowed unobtrusive monitoring during meals. Another smart eating utensil aimed at increasing user awareness of eating speed was proposed [74]. A summary of the measurement devices found in this review for metrics related to eating time and rate is provided in Table A5.

#### 3.3.6. Mass of Food Intake

##### Acoustic Sensors

Audio sensors, including internal and external microphones in a customized earbud, were used to develop a regression model for predicting the amount of consumed food [71]. Data from these microphones were analyzed to estimate intake, with a reported error of 35.4% when measuring food consumption using sensor and image features from annotations of 30 meals. A combination of motion and audio sensing leads to significantly more accurate estimates of food type (82.7% accuracy) than either modality alone.

##### Distance Sensor

A wearable sensor system, the Automatic Ingestion Monitor integrated with a ranging sensor, was used to estimate the dimensions of plates and bowls for portion size measurement [167]. The method was evaluated on a test bench using a calibrated protractor for positioning, considering three heights and three angles based on the natural behavior of participants in previous AIM-based studies. One major contribution of this study is that the model eliminates the need for fiducial markers, which require the users to carry around the references (checkerboards, blocks, and cards), and some require special dining setups, which increases the user burden.

##### Motion Sensors

Inertial sensors embedded in smartwatches were used in FluidMeter, an unobtrusive system for tracking fluid intake [168]. The system first distinguishes drinking activities from other movements, such as playing, running, and eating. It then analyzes sensor data from detected drinking episodes to identify micro-activities, including lifting the bottle, sipping, and releasing the bottle. Machine learning algorithms applied to features extracted during the sipping period estimate fluid intake per episode. Using data from 72 volunteers, FluidMeter achieved an overall fluid intake estimation error of 15%. Ultrasonic, RGB color, temperature, and accelerometer sensors were used to measure liquid intake and classify fluid type [73]. A computational framework applied machine-learning techniques for fluid intake classification, volume estimation, and bottle-state recognition. Results showed that regression-based volume estimation achieved a root-relative-squared error ranging from 1.12% to 13.36%.

##### Strain Sensors

A strain sensor combined with an acoustic sensor was used to estimate portion size through individually calibrated models based on Counts of Chews and Swallows (CCS models) [169]. Conducted in a laboratory setting, the study estimated chew and swallow counts using sensor signals and video recordings. CCS models were compared to diet diaries, showing lower reporting bias and error. The approach achieved a mean absolute percentage error of 32.2% ± 24.8% in portion size estimation. The sensor burden evaluated by a survey at the completion of the study indicated that chewing and swallowing sensors did not significantly affect the way subjects consumed their meals, suggesting that the recording burden can be significantly attenuated. A force-sensing resistor integrated into a smart tray was used in Mom’s Tray, a dietary monitoring system designed to measure the weight of ingested food [170]. The system incorporated prearranged RFID-tagged food packages and a mobile app to provide real-time feedback on food ordering and consumption in a school cafeteria setting.

##### Cameras

A deep learning-based image analysis system, PITA (Picture-to-Amount), was developed to predict the relative amount of each ingredient in a food image [61]. Extracted image features were used to estimate food weight through advanced boosting regression algorithms [171]. A dataset of 23,052 annotated images of Mediterranean cuisine, covering 226 dishes with a reference object for scale, was used to train the model. The proposed approach achieved a mean absolute percentage error of 3.73% between predicted and actual weight values.

##### Others

Universal Eating Monitor (UEM) with a concealed electronic balance was developed to enable covert, continuous weighing of a participant’s plate [172]. The effect of being aware that food intake is monitored by UEM on consumption amount was examined, with results indicating no significant impact on consumed mass [76]. A Mandometer, a device for continuous in-meal weight measurement, was used to develop an algorithm that extracts eating indicators such as total food intake mass and intake rate [75]. Recessed scales embedded in a table continuously measured plate weight at 15 Hz to track food consumption [173]. Bite weight was determined by the change in plate weight between each bite. A dining tray equipped with a video camera and three built-in weighing stations was used to monitor the weight of a bowl, plate, and drinking cup throughout a meal [174]. To evaluate image-based dietary assessment, food intake mass was recorded using a weight scale [175]. A summary of the measurement devices found in this review for metrics related to mass of food intake is provided in Table A6.

#### 3.3.7. Food Intake Environment

##### Cameras

A sensor device with an integrated camera was used to automatically capture images of a user’s meal surroundings to examine the dining environment [176]. The device was attached to a ceiling light in the user’s dining room to monitor meal settings. The methodology of finding a representative photograph captured during the mealtime was investigated by using the complexity of an image. A wearable camera, SenseCam, was used to assess the context of eating episodes in free-living conditions [80]. In the study, 40 adult participants wore the device while their diet was assessed through three image-assisted multiple-pass 24-h dietary recalls. Over 107 days, 742 eating episodes were analyzed, showing that most meals occurred at tables (27%) or sofas (26%), while standing (19%) or eating at desks (18%) was also common. Social interaction was observed in 45% of episodes, and media screens were viewed in 55% of cases. Meals consumed while watching television lasted 3.1 min longer and had higher energy intake compared to those without screen exposure. An egocentric wearable camera recorded over 33,000 images for the classification of food-related environments [177]. A hierarchical semantic classification approach categorized 15 distinct food-related scenes, achieving an accuracy of 56% and an F-score of 65%. A taxonomy of food-related environments that considers the main activities related to food (eating, cooking, buying, etc.) was prepared from the food-related environments. This semantic hierarchy aims to analyze the food-related activity at different levels of definition. This will allow for a better understanding of the user’s behavior. Built-in sensors, including a microphone on a smartphone or tablet and a microphone and accelerometer on a smartwatch, were used in FamilyLog, a dietary monitoring system for logging family mealtime activities [178]. The system automatically detected and recorded meal occurrences, duration, conversations, participants, and TV viewing. Evaluation using 77 days of data from 37 subjects across eight families demonstrated its effectiveness in unobtrusively capturing mealtime details. In addition to quantitative performance evaluation, user experience was also investigated by conducting 1-h interviews. The feedback from interviews shows that subjects felt uncomfortable recording and uploading acoustic and video data. Virtual reality (VR) technology was used to study the effect of the eating environment on food intake and behavior [179]. For that study, 15 adults consumed pizza rolls in two VR environments—a restaurant and an empty room—demonstrating VR’s potential as a tool for analyzing eating behavior in different settings. A neural network-based method, utilizing a two-stage training framework combining fine-tuning and transfer learning, was developed for automatic ingestion environment recognition [180]. Data from 30 participants wearing the AIM-2 sensor system, which features an egocentric wearable camera, were collected in free-living conditions. The proposed method addressed data imbalance challenges and achieved 96.63% classification accuracy across 22 different food environment classes, including restaurants, offices, dining rooms, kitchens, and cafeterias. Low-resolution infrared (IR) sensors combined with RGB video cameras were used to detect eating behavior and social presence in real-world settings [181]. While high-resolution cameras provided visual confirmation, limited battery life restricted continuous monitoring. Low-resolution IR sensors enabled automated detection but required validation for social and eating behavior tracking. By integrating both technologies, the system improved eating detection by 5% and social presence identification by 44% compared to video-only methods, demonstrating the effectiveness of multimodal sensing for automated eating environment monitoring.

## 4. Discussion

**Figure 3:**
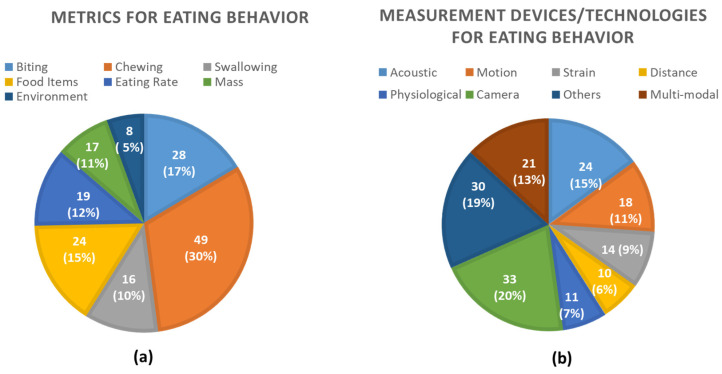
Summary of findings: (**a**) Number and percentage of articles found for quantifiable metrics of eating behavior; (**b**) Number and percentage of articles found for the measurement devices/technologies used to measure the eating behavior metrics.

This systematic review aimed to assess existing technology-driven devices for measuring eating behavior. To address RQ1, we identified and categorized quantifiable physiological and environmental metrics of eating behavior into seven major classes. To address RQ2, we grouped the devices and technologies used to capture these metrics into corresponding categories. Figure 3 summarizes the key findings. Among the identified metrics, chewing was the most frequently assessed physiological indicator (30% of studies), while camera-based systems were the most commonly used technology (20%).

**Figure 4:**
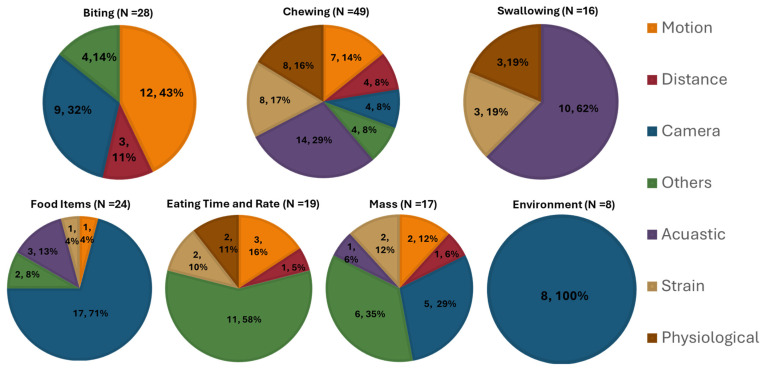
Summary of the number of studies related to individual eating behavior metrics and the technology used to measure eating behavior metrics.

Figure 4 summarizes the number of studies found in this review related to individual eating behavior metrics and the technologies used to measure these metrics. Out of the 161 reviewed full-text articles, 28 studies (17%) focused on the measurement of biting behavior using sensor-based approaches. Table A1 in Appendix A.1 presents a detailed summary of these studies. Among them, 43% (12 studies) employed motion sensors, such as accelerometers and gyroscopes; 11% (three studies) used distance-based sensors like proximity or ambient light sensors; and 32% (nine studies) adopted imaging technologies, including depth cameras, action cameras, and fisheye lenses. The remaining 14% (four studies) utilized other tools, such as weight sensors and smart forks. Notably, 36% (10 studies) evaluated their systems outside of laboratory conditions.

Chewing as a physiological eating behavior was assessed in 49 studies (30%) (Appendix A.2: Table A2). Among these, 29% (14 studies) used acoustic sensors, 14% (seven studies) relied on motion sensors, and 16% (eight studies) employed strain sensors. A diverse group of physiological sensors, including EMG and photoplethysmography (PPG), was used in 16% (eight studies). Distance sensors and imaging technologies (smartphone or digital cameras) were each used in 8% (four studies).

Swallowing behavior was explored in 16 studies (10%) (Appendix A.3: Table A3). Most of these (63%) used acoustic sensors for swallow detection. Strain sensors and physiological sensors (e.g., piezoelectric belts, RIP belts, EMG) were used in 19% (three studies) each. However, none of these systems were tested outside laboratory settings. Interestingly, 38% (six studies) offered real-time feedback to users based on swallow detection.

In terms of food item recognition, 24 studies (15%) were identified (Appendix A.4: Table A4). Acoustic sensors were used in only three studies, and motion or strain sensors in one study each. The majority of studies (17) relied on camera-based systems (e.g., smartphone, digital, or thermal cameras). Other sensor types included pH sensors, conductive sensors, and RGB color sensors for liquid food detection. None of these studies were tested in real-world settings, and only five studies (21%) provided real-time feedback.

Eating time and rate metrics were investigated in 19 studies (12%) (Appendix A.5: Table A5). Motion sensors were used in three studies, strain sensors in two, and optical sensors in one. Other tools, such as EMG, RIP belts, and smart utensils (e.g., Universal Eating Monitor, Smart Fork) were used in the remaining studies. Only three studies validated their methods in free-living environments.

Metrics related to food intake mass were examined in 17 studies (11%) (Appendix A.6: Table A6). Of these, one study used acoustic sensing and one used distance sensors. Motion sensors (accelerometers/gyroscopes) were used in two studies, and strain sensors in two. Imaging technologies were applied in five studies, and devices like weight scales and electronic balances were used in six studies. Only three studies assessed their systems in real-world conditions.

Our analysis reveals a strong link between the type of measurement device and the specific eating behavior metric it captures. For instance, acoustic sensors are predominantly used for detecting chewing (29% used acoustic) and swallowing (63% used acoustic) events due to their sensitivity to intraoral sounds. Motion sensors, including accelerometers and gyroscopes, are widely used to track biting (43% used motion) as they can detect subtle hand or jaw movements. In contrast, imaging technologies, such as smartphone cameras and depth sensors, are most frequently used for identifying food items (70% used camera), and eating environment (100% used camera), where visual data is essential. Similarly, strain sensors are used for contact-based detection of eating, such as swallowing, while weight-based systems (e.g., electronic scales) are applied to measure food intake mass over time. These pairings suggest that sensor selection is inherently tied to the physical nature of the behavior being measured. However, the feasibility of deploying these devices in real-world settings varies significantly. For example, wearable acoustic sensors can raise concerns regarding comfort and social acceptability, whereas camera-based systems require line-of-sight and often lack portability.

**Figure 5:**
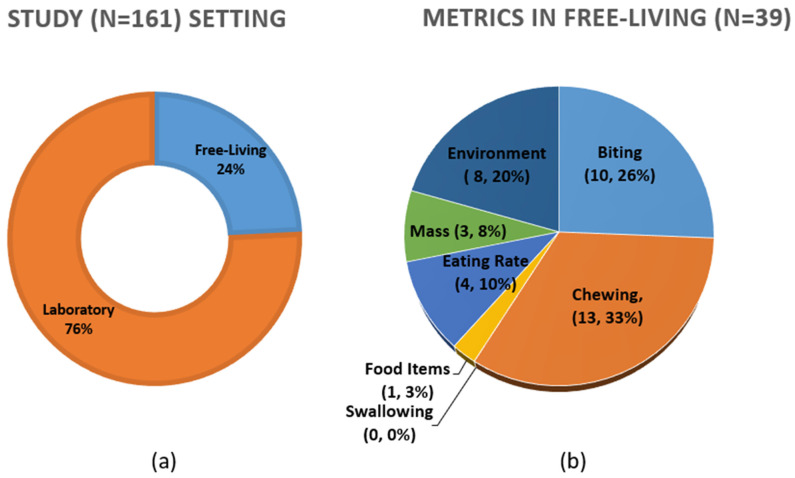
Summary of the study settings of the included studies. (**a**) Represents the percentage of included studies settings. (**b**) Represents the number (percentage) of measured eating behavior metrics in free-living setting.

Another critical finding of this review is the limited number of studies testing their systems outside laboratory settings. Figure 5 represents the summary of the study settings of the included studies in this review. Out of 161 studies analyzed, only 39 (24%) conducted evaluations in free-living environments. Among these 39 free-living studies, 13 (33%) studies were conducted to measure chewing-related eating behaviors. All eight studies related to eating environment were free-living settings, while zero (0%) studies were reported in free-living settings for swallowing-related eating behavior measurement.

In addition to analyzing the metrics and technologies used to measure eating behavior, it is equally important to consider the practical usability and social acceptability of these technologies, especially in non-laboratory, everyday environments. Despite the technical advancements observed across various studies, only a limited number evaluated user comfort or acceptability through structured feedback. Sensor burden, such as the discomfort or inconvenience caused by wearing certain devices, was mentioned in a few studies, often as a limiting factor for long-term adoption. For example, head-mounted cameras and facial electromyography sensors, although accurate, may intrude upon personal space or attract social attention, potentially discouraging their use in public settings. Likewise, throat-mounted sensors or bulky smart utensils may not integrate seamlessly into users’ daily lives. Among the reviewed studies, very few incorporated a formal user experience evaluation. Moreover, no study systematically evaluated how social contexts (e.g., eating with others, dining in restaurants) influence users’ willingness to wear or interact with these devices. These findings highlight a critical gap in current research: while sensor accuracy and detection capability are well documented, user-centered factors—including comfort, discretion, ease of use, and social integration—remain underexplored.

## 5. Challenges and Future Trends

Despite significant advancements in technology-driven methods for measuring physiological and environmental aspects of eating behavior over the past two decades, these methods are still not widely adopted in clinical and research settings. This review highlights that many existing solutions rely on stationary or portable sensor systems, which are restricted to specific locations and are therefore impractical for monitoring eating behavior in free-living conditions, where food consumption occurs in various settings throughout the day. In contrast, non-invasive wearable sensors offer a more practical approach for continuous monitoring in free-living environments. However, two key challenges must be addressed in wearable sensor technologies: sensor burden and privacy concerns. The issue of sensor burden raises critical questions regarding user comfort and acceptability. It is essential to evaluate whether a wearable device causes discomfort, restricts natural movement, alters eating behavior, or affects the user’s appearance. Unfortunately, most studies fail to assess these factors. To encourage widespread adoption, wearable sensors must be designed to be lightweight, unobtrusive, aesthetically acceptable, and seamlessly integrated into daily activities. Some studies have attempted to mitigate sensor burden by utilizing off-the-shelf devices such as smartwatches and Google Glass. However, while these solutions reduce the physical burden, they do not address privacy concerns. Privacy remains a major issue, particularly for acoustic-based and camera-based wearable sensors, as they continuously record sounds or capture images that may intrude on users’ personal lives. Further research is needed to develop privacy-preserving approaches, such as filtering out non-food-related sounds or images, to ensure user confidentiality and comfort [182]. Another critical finding of this review is the limited number of studies testing their systems outside laboratory settings. Out of 161 studies analyzed, only 30 (19%) conducted evaluations in free-living environments. Testing in real-world conditions is essential to validate usability and effectiveness in daily life. Furthermore, even in studies that tested systems in free-living conditions, experiments were often conducted with a small number of participants and for short durations, limiting the generalizability of findings. To assess the long-term impact of eating monitoring systems, studies should involve extended testing periods, such as weeks or months per user. A promising trend identified in this review is the increasing focus on enhancing accuracy through the integration of multiple sensor modalities and advanced machine learning algorithms, including deep learning. Of the 161 studies reviewed, 21 (13%) reported that performance improved when multiple sensor types were utilized. However, this progress is still in its early stages. Additionally, this review highlights a scarcity of studies quantifying liquid and beverage intake using technology. Only 10 (6%) of the analyzed studies reported quantifiable metrics for liquid or beverage consumption. Another indirect outcome of this review is the observation that, while many studies focus on detecting food intake and recognizing food types, they often fail to provide quantifiable metrics beyond detection. Eating behavior is highly dynamic and influenced by multiple contextual factors, including social settings (e.g., eating with family), physical environments (e.g., rural vs. urban food availability), and psychological conditions (e.g., stress levels). Integrating eating data from wearable sensors with additional technologies that capture these contextual variables could lead to the development of real-time behavioral models. These models, in turn, could inform personalized interventions to predict and modify obesity-related behaviors, ultimately contributing to improved public health outcomes.

## 6. Conclusions

This systematic review examined technology-driven approaches developed over the past two decades to detect and measure eating behavior metrics. The paper contributes a taxonomy of measurement devices for eating behavior, categorizing them based on physiological or environmental phenomena and sensor modalities. Current methodologies for detecting and measuring various aspects of eating behavior can be enhanced through the integration of multiple sensor modalities and the use of powerful machine learning algorithms, including pattern recognition, classification, regression, computer vision, and deep learning. Moreover, measurement devices need to be designed for use beyond the confines of laboratory settings. Addressing these challenges would enable the use of eating behavior monitoring devices in daily life, supporting the promotion of healthy eating habits.

## Figures and Tables

**Table 1:** Eligibility Criteria.

Inclusion Criteria	Exclusion Criteria
Articles published since 1 January 2000.	Articles not written in English
Articles published after peer-reviewed	Not an article, such as studies published as posters, abstracts, book chapters, database descriptions, and review articles
Articles must address a set of keywords including chewing, chewing rate, chewing frequency, biting, bite rate, bite frequency, swallowing, swallow rate, swallow frequency, eating rate, eating speed, meal duration, mealtime, eating amount, food items, portion size, mass of intake, eating environment, sensor, device, technology	Studies conducted on animals.
Articles that describe the measurement of quantifiable metrics for eating behavior after eating detection using technology.	Unrelated articles, such as studies that describe the eating detection process but with no quantifiable metrics for eating behavior, and studies that do not use technology to measure eating behavior.

**Table 2:** Search query strings to obtain candidate articles.

Search Strings	Databases					
	ACM	IEEE	PubMed	Science Direct	Scopus	Total
(chewing OR biting OR swallowing OR food items OR eating environment OR portion size) AND (sensor OR device OR technology)	60	252	35	454	135	936
(chewing rate OR chewing frequency OR bite rate OR bite frequency OR swallowing rate OR swallowing frequency) AND (sensor OR device OR technology)	114	125	78	301	231	849
(mealtime OR meal duration OR eating duration OR eating rate OR eating speed) AND (sensor OR device OR technology)	64	219	73	155	77	588

## Data Availability

Not applicable.

## Appendix A

### Appendix A.1

**Table A1:** Summary of the measurement devices for metrics related to biting.

Article	Device	Sensor	Position	Participants	Lab	Free Living	Real Time	Performance
[90]	Motion	Accelerometer, gyroscope	Wrist	22	Yes	Yes	No	F1 Score = 0.923
[38]	Motion	Orientation	Wrist	10	Yes	No	No	Sensitivity = 91%
[87]	Motion	Accelerometer Gyroscope	Wrist	271	Yes	Yes	No	Sensitivity = 75%
[88]	Motion	Accelerometer Gyroscope	Wrist	276	Yes	Yes	No	Accuracy = 79.7%
[44]	Motion	Gyroscope	Wrist	99	Yes	Yes	Yes	NA
[86]	Motion	Accelerometer Gyroscope	Wrist	34	Yes	Yes	No	False positive rate = 6.5% False Negative Rate = 3.3%
[84]	Motion	Accelerometer	Wrist	1	Yes	No	Yes	NA
[85]	Motion	Accelerometer Gyroscope	Wrist	3	Yes	Yes	No	Accuracy = 91.8%
[89]	Motion	Accelerometer Gyroscope	Wrist	12	Yes			F1 Score = 0.91
[91]	Motion	Smartwatch	Wrist	10	Yes	No	No	Mean Absolute Error = 3.99 g per bite
[183]	Motion	Gyroscope	Wrist	8	Yes	No	No	Accuracy ≥ 90%
[39]	Motion	Tri-axial accelerometer	wrist	15	Yes	No	No	Accuracy = 81.2%
[92]	Distance	Magnetic proximity	Neck	1	Yes	No	No	NA
[184]	Distance	Capacitive Sensing	3-D Printed Ice cream Cone	NA	Yes	No	Yes	NA
[93]	Distance	Ambient light	Neck	20	Yes	Yes	No	F1 Score = 77.1%
[95]	Camera	Depth Camera (Kinect Xbox One)	In front of the user	1	Yes	No	No	Sensitivity = 96.2%
[40]	Camera	SJ4000 Action Camera (Black)	3 feet away from the user	28	Yes	No	No	Accuracy = 85.4%
[185]	Camera	Digital camcorder	1.5 m away from the user	85	Yes	No	No	Recall = 91.7%
[94]	Camera	Digital Camcorder	1.5 m away from the user	85	Yes	No	No	F1 Score = 0.948
[96]	Camera	360 Degree Camera	In front of the table	4	Yes	Yes	No	Error = 26.2%
[97]	Camera	Digital camera	Ceiling	264	Yes	No	No	F1 score = 0.899
[99]	Camera	Digital camera	Ceiling	264	Yes	No	No	F1 score = 0.93
[98]	Camera	Digital Camera	In front of the table	18	Yes	No	No	Accuracy = 79%
[37]	Camera	Fisheye camera	Shoulder	16	Yes	Yes	No	NA
[186]	Others	Electrical Conductivity of Foods	Food Item	1	Yes	No	No	NA
[100]	Others	Augmented Fork	Fork	141	Yes	Yes	Yes	NA
[101]	Others	Weight Sensors	Plate	24	Yes	Yes	No	Precision = 0.76 Recall = 0.76
[187]	Others	Voltage Divider	Embedded Fork	6	Yes	Yes	No	Accuracy = 77%

### Appendix A.2

**Table A2:** Summary of measurement devices for metrics related to chewing.

Article	Device	Sensor	Position	Participants	Lab	Free Living	Real Time	Performance
[111]	Acoustic, Motion	Accelerometer, Gyroscope, Microphone	Earbud	5	Yes	No	No	Accuracy = 97%
[103]	Acoustic, Camera	Microphone, camera	Ear	6	Yes	Yes	No	Accuracy = 80%
[104]	Acoustic	Throat Microphone	Neck	12	Yes	No	No	Accuracy = 86.6%
[113]	Acoustic	Two-channel condenser microphone	Under ear	18	Yes	No	No	F score = 0.8
[105]	Acoustic	Throat microphone	Neck	12	Yes	No	No	F score = 71.4%
[114]	Acoustic, Motion	9 axis IMU, microphone	Wrist, Ear	6	Yes	Yes	No	Recall = 84% Precision = 67%
[108]	Acoustic	Throat Microphone	Neck	8	Yes	No	No	Accuracy = 0.783
[102]	Acoustic	Microphone	Ear	NA	Yes	No	No	Error Rate = 1.93%
[147]	Acoustic	Ultrasonic Doppler Sensor	Neck	10	Yes	No	No	Accuracy = 91.4%
[10]	Acoustic	Microphone	Ear	55	Yes	No	No	Precision > 80 Recall > 80%
[106]	Acoustic	Bluetooth Headset	Ear	28	Yes	No	No	Accuracy = (77–94)%
[112]	Acoustic	Skin contact microphone	Neck	14	Yes	No	No	F score = 77.5%
[109]	Acoustic	Bone conduction microphone	Ear	6	Yes	No	Yes	Accuracy = 97.6%
[107]	Acoustic, Motion	Microphone, 9-axis IMU, 9 axis motion sensor	Ear, Wrist, Head	6	Yes	Yes	No	Accuracy = 85%
[110]	Acoustic	Bone conduction microphone	Ear	9	Yes	Yes	No	Accuracy = 97.1%
[115]	Acoustic	Microphone	Eyeglass	5	Yes	No	No	F score = 0.96
[119]	Motion, physiological	Accelerometer, Orientation, Gyroscope, EMG	Wrist	36	Yes	No	No	F score = 0.92
[188]	Motion, Strain	Accelerometer, Hand Gesture sensor, Piezoelectric strain	Wrist, Wrist, Below Ear	12	Yes	Yes	No	Accuracy = 89.8%
[118]	Motion	Single axis accelerometer	Temporalis Muscle	4	Yes	No	No	Accuracy = 97% F score = 93%
[116]	Motion	3 axis accelerometer, 3 axis gyroscope, 3 axis magnetometer	Chin	13	Yes	No	No	NA
[120]	Motion	IMU	Ear	8	Yes	No	No	F score = 0.91
[121]	Strain	Piezoelectric Strain	Below ear	20	Yes	No	No	Accuracy = 80.98%
[122]	Strain	Jaw motion sensor	Below ear	12	Yes	Yes	No	Accuracy = 86.86 ± 6.5%
[48]	Strain	Piezoelectric Strain Sensor	Below Ear	30	Yes	No	No	Error rate = 9.66%
[123]	Strain	Piezoelectric Strain Sensor	Below ear	5	Yes	No	No	Error rate = 8.09%
[49]	Strain	Piezoelectric Strain Sensor	Below Ear	30	Yes	Yes	No	Error rate = 15.01%
[124]	Motion, Strain	Accelerometer, Piezoelectric Strain	Eyeglass temple, below ear	10	Yes	No	No	F score = 99.85%
[125]	Motion, Strain	Accelerometer, hand gesture sensor, Piezoelectric Strain	Wrist, Wrist, Below Ear	12	Yes	Yes	No	Accuracy = 93%
[47]	Strain	Piezoelectric Strain	Temporalis Muscle	10	Yes	Yes	No	Error Rate = 3.83%
[126]	Physiological, Strain	EMG, piezo	Chin, Neck	10	Yes	No	No	Accuracy = 0.938
[93]	Distance	Proximity	Neck	20	Yes	Yes	No	F1 Score = 77.1%
[50]	Motion, Distance	Accelerometer, Infrared Distance Sensor	Ear pinna	22	Yes	No	No	Precision = 85.3% Recall = 84.5%
[127]	Distance	Proximity Sensor	Necklace	32	Yes	Yes	No	Precision = 78.2&% Recall = 72.5%
[128]	Distance	Proximity Sensor	Right temple of eyeglass	10	Yes	No	No	Error rate = 2.69%
[129]	Distance	Proximity Sensor	Eyeglass	20	Yes	No	No	Accuracy = 96.4%
[130]	Physiological	EMG	Right and Left masseter, anterior temporalis muscle	37	Yes	No	No	NA
[52]	Physiological	EMG	Right and left masseter, Right and left temporalis muscle	13	Yes	No	No	NA
[131]	Physiological	Portable EMG	Center of masseter, mastoid	28	Yes	No	No	NA
[132]	Physiological	EMG	Eyeglass	8	Yes	No	No	Precision = 80%
[65]	Acoustic, Physiological	Microphone, photoplethysmography (PPG)	Ear	22	Yes	Yes	No	Accuracy = 0.938
[135]	Physiological	EMG	Between the mastoid and the masseter muscle	15	Yes	Yes	No	Sensitivity > 90%
[133]	Physiological	EMG	Eyeglass	10	Yes	Yes	No	Precision > 77%
[136]	Physiological	Myoelectric sensor	Masseter muscle	8	Yes	No	Yes	NA
[51]	Camera	Digital camera	In front of user	6	Yes	No	No	NA
[40]	Camera	SJ4000 Action Camera (Black)	3 feet away from user	28	Yes	No	No	Accuracy = 88.9%
[137]	Camera	Digital camera	In front of the user	37	Yes	No	No	NA
[138]	Camera	Smartphone camera	In front of the user	100	Yes	No	No	Error Rate = 7%
[174]	Camera	video camera	Dining tray	NA	Yes	No	No	NA
[139]	Others	EMG, Piezoelectric Strain Sensor, Piezoresistive Sensor, Pressure Sensor	Right temporalis muscle, left temporalis muscle, eyeglass, ear canal	15	Yes	No	No	NA

### Appendix A.3

**Table A3:** Summary of measurement devices for metrics related to swallowing.

Article	Device	Sensor	Position	Participants	Lab	Free Living	Real Time	Performance
[140]	Acoustic	Throat microphone	Throat	21	Yes	No	No	NA
[12]	Acoustic	Throat microphone	Throat	20	Yes	No	No	Accuracy = 84.7%
[141]	Acoustic	Throat and Ambient Microphone	Throat	7	Yes	No	No	Recall > 85%
[142]	Acoustic	Throat Microphone	Throat	7	Yes	No	No	Accuracy > 94%
[143]	Acoustic	Microphone	Neck	85	Yes	No	Yes	Accuracy > 79.3%
[104]	Acoustic	High fidelity microphone	Neck	12	Yes	No	Yes	Accuracy = 86.6%
[147]	Acoustic	Ultrasonic Doppler Sensor	Neck	10	Yes	No	No	Accuracy = 78.4%
[108]	Acoustic	Throat Microphone	Neck	8	Yes	No	No	Accuracy = 0.712
[110]	Acoustic	Bone conduction microphone	Neck	9	Yes	No	Yes	Accuracy = 97.1%
[109]	Acoustic	Bone conduction microphone	Ear	6	Yes	No	Yes	Accuracy = 97.6%
[144]	Strain	Piezoelectric Sensor	Lower Trachea	20	Yes	No	Yes	F score = 80%
[189]	Strain	Piezoelectric Sensor	Lower Trachea	20	Yes	No	Yes	F score = 91.2%
[55]	Strain	Piezoelectric Sensor, IMU	Neck	10	Yes	No	No	F score = 76.07%
[145]	Physiological	Piezo-respiratory belt	Chest	3	Yes	No	No	Accuracy = 80%
[146]	Physiological	Respiratory Inductance Plethysmography (RIP) belt	Chest and Abdomen	6	Yes	No	No	Precision = 80%
[46]	Physiological	EMG	Masseter muscle	16	Yes	No	No	F1 score = 0.87

### Appendix A.4

**Table A4:** Summary of measurement devices for metrics related to food items.

Article	Device	Sensor	Position	Participants	Lab	Free Living	Real Time	Performance
[104]	Acoustic	High fidelity microphone	Neck	12	Yes	No	Yes	Accuracy = 84.9%
[144]	Strain	Piezoelectric Sensor	Lower Trachea	20	Yes	No	Yes	Precision = 80%
[148]	Acoustic	Lavalier microphone (MAONO)	Shirt collar	10	Yes	No	No	F score = 97.2%
[149]	Acoustic	Microphone	Over Ear	16	Yes	Yes	No	F score = 97.44%
[71]	Motion	Internal and external microphone, 9-axis IMU in the wrist, 9-axis IMU in head	Ear, Wrist, Head	6	Yes	No	No	Accuracy = 82.7%
[190]	Camera	Digital camera	On top of table	NA	Yes	No	No	NA
[58]	Camera	Digital camera	On top of table	NA	Yes	No	No	NA
[150]	Camera	Cellular phone camera	Smartphone	NA	Yes	No	No	Accuracy = 61.34%
[176]	Camera	Digital camera	Ceiling	NA	Yes	No	No	NA
[57]	Camera	Smartphone camera	Smartphone	NA	Yes	No	Yes	Accuracy = 81.55%
[18]	Camera	Smartphone camera	Smartphone	NA	Yes	No	No	Accuracy = 92.1%
[151]	Camera	Smartphone camera	Smartphone	NA	Yes	No	No	NA
[152]	Camera	Slight tilt in front of user	Smartphone	5	Yes	No	Yes	Classification rate = 74.8%
[153]	Camera	Smartphone camera	Smartphone	NA	Yes	No	Yes	Accuracy = 88.5%
[154]	Camera	Thermal Camera	Smartphone	NA	Yes	No	No	Accuracy = 88.93%
[155]	Camera	Color, Thermal Cameras	Wrist	NA	Yes	No	No	NA
[156]	Camera	Camera	Smartphone	NA	Yes	No	No	Accuracy = 95%
[157]	Camera	Smartphone camera	Smartphone	NA	Yes	No	No	NA
[158]	Camera	Smartphone camera	Smartphone	NA	Yes	No	No	NA
[60]	Camera	Smartphone camera	Smartphone	NA	Yes	No	No	Accuracy = 82%
[59]	Camera	Smartphone camera	Smartphone	NA	Yes	No	No	Accuracy = 87.3%
[159]	Camera	Smartphone camera	Smartphone	NA	Yes	No	No	NA
[160]	Others	Ion selective pH, conductivity	Cup	NA	Yes	No	No	Accuracy = 79%
[73]	Others	Ultrasonic, RGB color, Temperature	Bottle	NA	Yes	No	No	Accuracy = (74.93–94.98%)

### Appendix A.5

**Table A5:** Summary of measurement devices for metrics related to eating time and rate.

Article	Device	Sensor	Position	Participants	Lab	Free Living	Real Time	Measured Metric
[84]	Motion	Accelerometer	Wrist	1	Yes	No	Yes	Eating speed
[126]	Strain	Piezoelectric	Neck	10	Yes	No	Yes	Eating speed
[155]	Physiological	EMG	Wrist	17	Yes	Yes	No	Eating speed
[66]	Motion	Accelerometer, IMU	Wrist	NA	Yes	No	Yes	Eating speed
[161]	Motion	IMU	Wrist	36	Yes	No	No	Eating Speed
[65]	Strain	Piezoelectric Strain	Temporalis Muscle	12	Yes	No	No	No. of meals, meal duration, Duration of actual ingestion
[67]	Distance	Optical Sensor	Ear	11	Yes	Yes	No	Mealtime
[162]	Physiological	Two Respiratory Inductance Plethysmography (RIP) belts	Chest and abdomen	14	Yes	No	No	Mealtime, duration
[64]	Others	Universal Eating Monitor	Table	60	Yes	Yes	No	Eating rate
[63]	Others	Weight Scale	Table	35	Yes	No	No	Eating rate
[42]	Others	Sussex Ingestion Pattern Monitor	Table	35	Yes	No	No	Eating rate, bite size, meal duration
[163]	Others	Smart Fork	Fork	11	Yes	Yes	Yes	Eating speed
[164]	Others	Glucose sensor	Artificial pancreas	30	Yes	No	No	Meal-size
[165]	Others	Smart Fork	Fork	128	Yes	No	No	Eating rate
[191]	Others	Smart utensil	Utensil	NA	Yes	No	No	Eating rate
[166]	Others	Pressure Sensor	Sheet	2	Yes	No	No	Mealtime, pace, duration
[74]	Others	Smart utensil	Utensil	10	Yes	No	No	Eating rate

### Appendix A.6

**Table A6:** Summary of measurement devices for mass of food intake.

Article	Device	Sensor	Position	Participants	Lab	Free Living	Real Time	Performance
[71]	Acoustic	Internal and external microphone, 9-axis IMU in wrist, 9-axis IMU in head	Ear, Wrist, Head	6	Yes	No	No	Error = 35.4%
[73]	Motion	Accelerometer	Bottle	NA	Yes	No	No	Error = 13.36%
[167]	Distance	Time of Flight (ToF)	Eyeglass	NA	Yes	No	No	NA
[168]	Motion	Gyroscope, Accelerometer	Wrist	41	Yes	Yes	No	Accuracy = 59.2%
[170]	Strain	Force sensing resistor	Tray	10	Yes	Yes	No	NA
[192]	Strain	Piezoelectric Strain	Temporalis Muscle	18	Yes	Yes	No	NA
[61]	Camera	Smartphone camera	Smartphone	NA	Yes	No	No	NA
[171]	Camera	Smartphone camera	Smartphone	NA	Yes	No	No	Error = 3.73%
[172]	Others	Electronic Balance	Table	26	Yes	No	No	NA
[76]	Others	Electronic Balance	Table	39	Yes	No	No	NA
[75]	Others	Mandometer	Table	77	Yes	No	No	Accuracy = 0.69%
[173]	Others	Weight scale	Table	72	Yes	No	No	NA
[175]	Others	Weight scale	Table	84	Yes	No	No	NA
[174]	Others	Weight scale	Tray	NA	Yes	No	No	NA
